# HPLC Analysis of Supercritical Carbon Dioxide and Compressed Propane Extracts from *Piper amalago* L. with Antileishmanial Activity

**DOI:** 10.3390/molecules17010015

**Published:** 2011-12-22

**Authors:** Vanessa da Silva Carrara, Lara Zampar Serra, Lúcio Cardozo-Filho, Edézio F. Cunha-Júnior, Eduardo C. Torres-Santos, Diógenes Aparício Garcia Cortez

**Affiliations:** 1 Departamento de Farmácia, Universidade Estadual de Maringá, 87020-900, Maringá, Paraná, Brazil; 2 Departamento de Engenharia Química, Universidade Estadual de Maringá, 87020-900, Maringá, Paraná, Brazil; 3 Laboratório de Bioquímica de Tripanossomatídeos, Instituto Oswaldo Cruz, FIOCRUZ, Rio de Janeiro, 21040-360, Brazil

**Keywords:** *Piper amalago* L., pyrrolidine alkaloid, supercritical carbon dioxide, HPLC validation, antileishmanial activity

## Abstract

*Piper amalago* L. leaves were extracted with supercritical carbon dioxide and compressed propane under different conditions, and with chloroform by the conventional maceration method. These methods were compared for the pyrrolidine alkaloid content. Supercritical carbon dioxide (SFE-CO_2_) at 313 K and 12.55 MPa showed the highest selectivity for the main compound (600.53 mg/g of extract). A gradient high-performance liquid chromatography (HPLC) method was developed and validated to quantify the alkaloid *N*-[7-(3′,4′-methylenedioxyphenyl)-2(Z),4(Z)-heptadienoyl]pyrrolidine (**1**) in the extracts. The HPLC method showed linearity, precision and accuracy, allowing the quantitative analysis of the alkaloid in all the samples. All the extracts were tested against the promastigote and intracellular amastigote forms of *Leishmania amazonensis*. The antileishmanial activity was evaluated in terms of inhibitory concentration for 50% of protozoa (IC_50_). The cytotoxicity was also evaluated against J774A1 macrophages, and the cytotoxic concentrations for 50% of macrophages were obtained (CC_50_). The SFE-CO_2_ (313 K; 12.55 MPa) extract showed the highest antileishmanial activity with the following IC_50_ values of 16 and 7 µg/mL against the promastigotes and intracellular amastigotes forms, respectively. The extract showed low cytotoxicity with a CC_50_ value of 93 µg/mL.

## 1. Introduction

Leishmaniasis affects about 12 million people in the tropical and subtropical areas of the World, being a serious health public problem [1]. The urban development, migration of people to endemic areas and human immunodeficiency virus infection are the main factors for the increasing of infected individuals [2]. This disease is caused by protozoans of the genus *Leishmania* that lead to cutaneous, visceral, and mucosal clinical forms [3,4].

Pentavalent antimonials, amphotericin and pentamidine are still the first choice drugs for the treatement of leishmaniasis, although they require long-term treatment, stimulate drug resistance, and are toxic. Miltefosine is a novel compound active against leishmaniasis, but it is teratogenic [5]. These facts indicate a need to discover new drugs, and plants have been considered as potential sources of new and more effective products [6].

The species of the genus *Piper* (*Piperaceae*) are used in folk medicine for the treatment of many diseases. Alkaloids which are related to different biological properties analgesic, anti-inflammatory, antitumor, anxiolytic, antidepressant, immunomodulatory, inhibition of cholesterol acetyltransferase and antimicrobial activities have been found in several species, such as *Piper longum*, *Piper sarmentosum*, *Piper nigrum*, *Piper ovatum*, *Piper hispidum*, *Piper arboreum*, *Piper tuberculatum* [7,8,9,10,11,12,13,14,15,16].

*Piper amalago* L. roots have been studied chemically and the presence of sesquiterpenes, pyrrolidine, and isobutylamides identified [17,18,19,20,21]. Pyrrolidine amides have been isolated from *P. amalago* L. leaves, which showed important activity against *L. amazonensis* [22].

The extraction of some materials using compressed gases in supercritical states has been investigated over the past 20 years within the food, cosmetics and pharmaceutical industries, due to the legislative restrictions, which require the elimination of the solvent residues in their products [23,24,25,26]. Supercritical fluid extraction employing carbon dioxide (SFE-CO_2_) has been chosen to extract components of low to medium polarity from solid and liquid pharmaceutical matrices, due to its safety, availability and low cost [27]. Several alkaloids have been extracted by this method, such as piperine, purine, pyrrolizidine, isoquinoline, quinolizidine, indole, and tropane alkaloids [28,29,30,31,32,33,34,35,36]. SFE-CO_2 _offers advantages over the traditional methods, since there is no use of organic solvents, generating only the extract or substance of interest. Supercritical CO_2_ can solubilize the analytes because its diffusion properties are similar those of gases, and its solvation power is similar to pentane. Moreover, its selectivity can be modulated by controlling the pressure and temperature, in order to obtain good yields of the isolated compounds in a short period of time. Thus, the additional purification steps are not necessary [37,38]. This technique also has a high sample load capacity and allows faster analysis even in complex samples. Furthermore, it can be carried out under mild conditions and preserves the thermolabile substances, and those subject to hydrolysis and hydrosolubilization. Therefore, the matrix and the extracts are not exposed to harmful solvents, and they are protected against degradation by chemical reactions caused by light, heat and oxygen [39,40].

Plant extracts have been analysed by HPLC method, which is much explored for control quality of phytochemicals [41,42,43,44,45,46]. Analytical methods need to be validated in order to ensure the efficacy, safety, and quality of medicinal products, complying with regulatory requirements of the drug registration. The purpose of an analytical method validation is to ensure that each measurement in routine analysis will be close enough to the actual value for the compound content in the sample [47,48].

The objective of the present study was to develop and validate a HPLC method in order to quantify the pyrrolidine alkaloid with important antileishmanial activity in the extracts of *P. amalago* L. leaves. Extractions employing supercritical carbon dioxide, compressed propane, and chloroform were compared in terms of alkaloid content, using the validated HPLC method. The extracts were tested against the promastigote and intracellular amastigote forms of *L. amazonensis*.

## 2. Results and Discussion

### 2.1. HPLC Method Development

The chromatographic profile was obtained in order to quantify only the compound *N*-[7-(3′,4′-methylenedioxyphenyl)-2(Z),4(Z)-heptadienoyl]pyrrolidine (**1**) present in the samples, through eluent and flow rate optimization. Thus, different contents of acetonitrile-water (with 1% acetic acid), gradients and isocratic systems and flow rates (0.5, 0.8 and 1.0 mL/min) were tested [44]. The combination of acetonitrile-water (with 1% acetic acid) 58:42 ratio (v/v) at a flow rate of 1 mL/min, at 298 K for 10 min, and 100% of acetonitrile from 11 to 15 min, resulted in a good separation. The detection wavelengths for the alkaloids were previously determined in a Thermo Fisher Scientific Model Evo 60 spectrophotometer (MA, USA) with Thermo Scientific Vision Lite^TM^ version 4.0 software (MA, USA), and the maximum absorption was found to occur at 260 nm.

**Figure 1:**
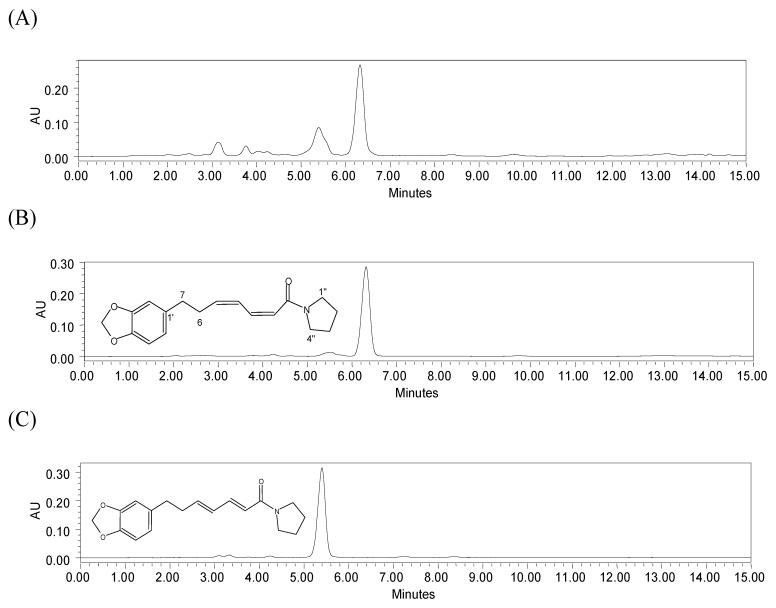
HPLC chromatograms of (**A**) chloroform leaf extract of *P. amalago* L.; (**B**) compound (**1**) (R_t_ = 6.3 min) and (**C**) compound (**2**) (R_t_ = 5.4 min). Chromatographic conditions: YMC Pack Pro C18 column; mobile phase: acetonitrile-water (with 1% acetic acid) (58:42 v/v) for 10 min, and 100% of acetonitrile from 11 to 15 min; flow rate 1 mL/min; temperature 298 K; detection at 260 nm.

The chromatograms of the chloroform extract and the two major alkaloids isolated from this plant are shown in Figure 1. The chromatographic profiles of the compounds **1** and **2** showed well-resolved peaks, with retention times (R_t_) of 6.3 and 5.4 min, respectively. The other peaks in the chloroform extract corresponded to unknown compounds.

### 2.2. Validation

To verify whether the HPLC method was appropriate for the quantification the compounds of interest, the following data were evaluated: linearity, precision, limits of detection and quantification, and accuracy. The validation was performed according to resolution RE n. 899, 2003 [49]. Compound **1** was used as the standard because it is the major compound in *P. amalago* L. With the use of this technique it was possible to detect and quantify the major alkaloid, because it presented linearity, precision and accuracy values within the study ranges. Thus, it complied with regulatory requirements for the reliable analysis of compounds in extracts.

#### 2.2.1. Linearity

Linear regression analysis was used to calculate the validation parameters of the calibration curve. Good linearity was observed in the range of 23.05 to 184.4 μg/mL. The regression equation of the calibration curve was y = 38942x − 25.71, with the correlation coefficient (r^2^) of 0.9988.

#### 2.2.2. Precision

The method precision was evaluated through studying of the repeatability and intermediate precision on three non-consecutive days, with triplicate analysis at three concentrations (23.05, 92.2 and 184.4 μg/mL) (Table 1). The results are agreement with those reported in the literature, since these phytochemicals presenting RSD values below 5% [50].

#### 2.2.3. Accuracy

The accuracy was determined using the recovery test. The recovery data were obtained from the relationship between the amount of standard added and the amount detected (Table 1). The RSD was lower than 15%, as expected for a complex sample [50].

**Table 1:** Repeatability, intermediate precision, and accuracy data of the method for the standard determination by HPLC.

Alkaloid	Analyte concentration (μg/mL)	Repeatability (RSD%)	Intermediate precision(RSD%) ^1^	Recovery (%) (mean ± SD ^2^)	Mean ± SD	RSD (%)
Compound 1	23.05	1.34	2.29	103.00 ± 3.19	102.91 ± 0.94	0.91
	92.2	2.27	1.13	101.93 ± 2.04		
	184.4	0.83	2.50	103.80 ± 3.10		

^1^ RSD is the relative standard deviation for each sample (n = 3); ^2^ SD is the standard deviation.

#### 2.2.4. Limits of Detection and Quantification

The limit of detection, in other words, the lowest amount of analyte which can be detected, but not quantified in the sample was found to be 4.35 μg/mL. The limit of quantification, defined as the lowest concentration of a standard which can be quantified with acceptable precision and accuracy was determined as 14.49 μg/mL.

### 2.3. Analysis of Alkaloid Content in Extracts Obtained by Supercritical CO_2_, Compressed Propane and Chloroform

**Figure 2:**
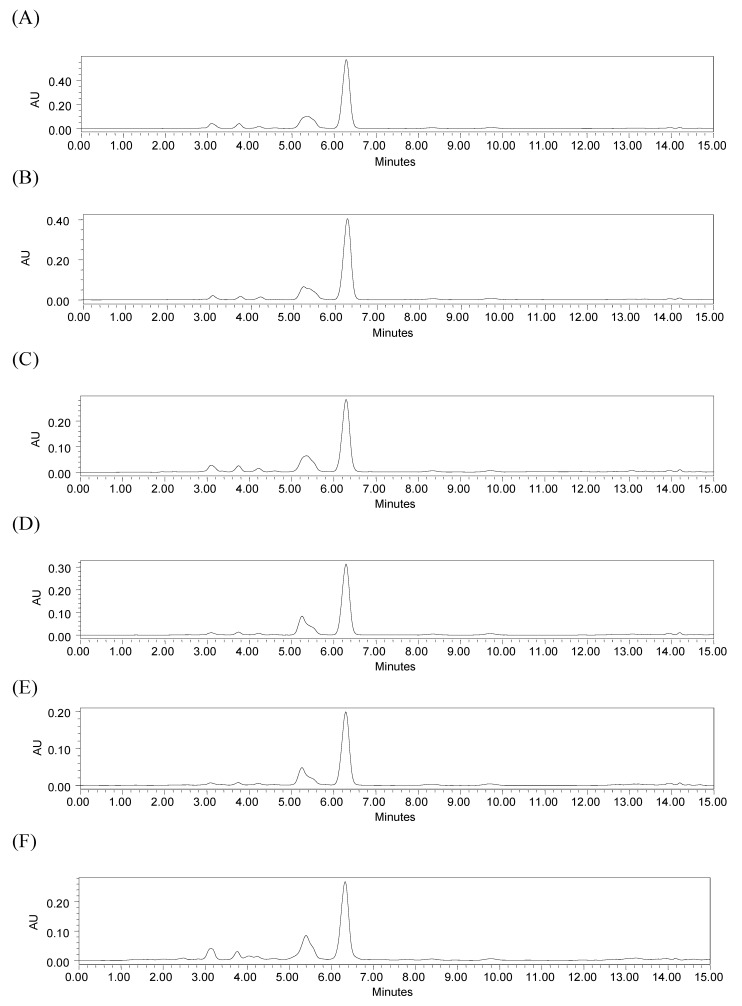
HPLC chromatograms of extracts: (**A**) SFE-CO_2_ (313 K, 12.55 MPa); (**B**) SFE-CO_2_ (333 K, 20.5 MPa); (**C**) Compressed propane (293 K, 15.0 MPa); (**D**) Compressed propane (313 K, 15.0 MPa); (**E**) Compressed propane (333 K, 15.0 MPa); (**F**) chloroform extract obtained by maceration. Chromatographic conditions: YMC Pack Pro C18 column; mobile phase: acetonitrile-water (58:42 v/v) with 1% acetic acid for 10 min, and 100% of acetonitrile from 11 to 15 min; flow rate 1 mL/min; temperature 298 K; detection 260 nm.

Supercritical CO_2_ and compressed propane extractions under different conditions were evaluated for alkaloid content by HPLC and compared with traditional maceration extraction using chloroform. The extractions showed similar chromatogram profiles (Figure 2). It was possible to quantify the content of compound **1** (R_t_ 6.3 min) in all samples from the regression equation. Despite the fact that the chromatographic profile of compound **2** showed a well-resolved peak, as indicated in Figure 1, it was not quantified, because its corresponding peak did not appear well-resolved in the extract chromatograms (Figure 2).

The contents of the alkaloid **1** in the samples expressed as mg/g of extract and mg/g of dried plant were calculated using the following formulas:


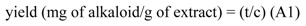


where t = alkaloid content (mg) in the extract solution at 0.3 mg/mL; c = extract concentration at 0.0003 g/mL, and:


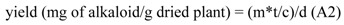


where m = mass of the extract obtained after total extraction time; t = alkaloid content (mg) in the extract solution at 0.3 mg/mL; c = extract concentration at 0.3 mg/mL, d = dried plant amount.

**Figure 3:**
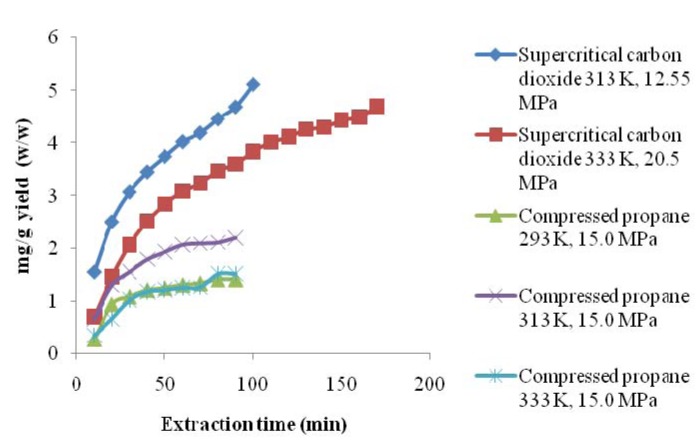
The cummulative yield of compound **1**, with respect to extraction time, using supercritical CO_2_ and compressed propane.

The conventional extraction method of maceration with chloroform led to the highest yield of compound **1** in the dried plant. SFE-CO_2_ at 313 K and 12.55 MPa and SFE-CO_2_ at 333 K and 20.5 MPa provided similar yields. SFE-CO_2_ (313 K; 12.55 MPa and 333 K; 20.5 MPa) indicated a higher alkaloid content in the dried plant than compressed propane (293, 313, 333 K; 15.0 MPa) (Table 2). SFE-CO_2_ at 313 K and 12.55 MPa was the more effective extraction method than compressed propane, because extracted high content of the alkaloid in less time (Figure 3). The compressed propane at 313 K and 15.0 MPa showed higher yield than compressed propane at 293 K, 333 K, and 15.0 MPa. There was no significant difference in the yields of this compound in the dried plant obtained using compressed propane at 293 K, 333 K, and 15.0 MPa.

**Table 2:** Alkaloid content in *P. amalago* L. samples, expressed in mg of alkaloid/g of extract (w/w), and in mg of alkaloid/g of dried plant (w/w).

Extraction Method	mg of alkaloid/g of extract (w/w)	mg of alkaloid/g dried plant (w/w)
	mean ± SD (n = 3)	mean ± SD (n = 3)
SFE-CO_2_	600.53 ± 21.08	5.11 ± 0.18 ^b^
313 K and, 12.55 MPa		
SFE-CO_2_	454.63 ± 18.93	4.70 ± 0.19 ^b^
333 K and, 20.5 MPa		
Compressed propane	296.06 ± 11.23 ^a^	1.40 ± 0.05 ^c^
293 K and, 15.0 MPa		
Compressed propane	345.56 ± 10.45	2.20 ± 0.06
313 K and, 15.0 MPa		
Compressed propane	209.60 ± 4.55	1.51 ± 0.03 ^c^
333 K and, 15.0 MPa		
Maceration with chloroform	306.83 ± 5.82 ^a^	12.96 ± 0.24

SD = standard deviation; Means followed by same lowercase letters did not differ statistically (Tukey test, *p* > 0.05); w/w = weight/weight.

The contents of alkaloid **1** in the extracts are shown in Table 2. The extracts obtained using compressed propane at 293 K and, 15.0 MPa did not differ statistically from the chloroform extract. Compressed propane at 313 K, and 15.0 MPa extracted purer alkaloids in a shorter time than the chloroform method. SFE-CO_2_, mainly at 313 K and, 12.55 MPa, extracted a higher purity alkaloid than compressed propane and chloroform, as can be observed in Table 2 and Figure 2. The peak area of compound **1** shown in n chromatogram (A) for the SFE-CO_2_ (313 K; 12.55 MPa) extract was higher than that shown in chromatogram (F) for the chloroform extract, indicating a higher content of the alkaloid in the SFE-CO_2_ (313 K; 12.55 MPa) extract and the better selectivity of the corresponding method. The ^1^H-NMR spectral data of the SFE-CO_2 _(313 K; 12.55 MPa) extract also showed higher purity for the alkaloid than chloroform extract. Besides the high purity of the alkaloid obtained, it is important to consider that the time required for the SFE-CO_2 _was around 80 min, while for the maceration method using a potentially harmful solvent the total time was five days for the complete procedure. Therefore, although extraction using chloroform as the solvent leads to high yields from the dried plant mass, it is a potentially harmful procedure and showed lower selectivity than SFE-CO_2_. This indicates that the SFE-CO_2_ (313 K; 12.55 MPa) extract would need fewer purification steps than the chloroform extract. Thus, SFE-CO_2_ at 313 K and 12.55 MPa is an economically attractive alternative for the extraction of pyrrolidine alkaloids. It is interesting to note that the SFE-CO_2_ method offers significant selectivity for compound **1** without the use of modifier, leading to a product rich in a desired compound without organic residues. In general, studies report the application of modifiers to improve the content of different alkaloids [28,29,30,31,32,33,34,35,36]. To our knowledge, the extraction of *N*-[7-(3′,4′-methylenedioxyphenyl)-2(Z),4(Z)-heptadienoyl]pyrrolidine (**1**) using SFE-CO_2_ and compressed propane has not been previously reported in the literature.

### 2.4. Antileishmanial Activity of the Extracs Against the Promastigotes and Intracellular Amastigotes Forms

The extracts were evaluated for the antileishmanial activity. The SFE-CO_2_ (313 K; 12.55 MPa) extract containing the highest alkaloid content was the most active against the amastigote forms of *Leishmania*, and it showed the highest SI. The anti-promastigote activity of the SFE-CO_2 _(313 K; 12.55 MPa) extract did not differ statistically of the chloroform extract and compressed propane (313 K, 15.0 MPa). The results obtained through the experiments were significant compared to the control group, with p < 0.05 (Student *t* test) (Table 3). Compounds **1** and **2** in the SFE-CO_2 _extract may be the main components of *P. amalago* L. leaves responsible for the biological activity [22]. However, the biological role of other compounds in the extract needs to be researched.

The nitric oxide production of macrophages treated with the most active extract against the amastigote forms was evaluated to determine whether anti-amastigote activity resulted from activation of this antileishmanial mechanism. The SFE-CO_2 _(313 K; 12.55 MPa) extract did not act on the production of nitric oxide, suggesting a direct and selective action on the intracellular amastigotes. A detailed evaluation about the mechanism of action is needed. Medicines selective in killing amastigotes are more efficient to combat human leishmaniasis, since amastigotes are the parasitic forms that persist in the infected host [51].

**Table 3:** Antipromastigote and anti-amastigote activity, cytotoxicity to the J774A1 macrophages, and SI of the extracts of *P. amalago* L. leaves and pentamidine isethionate (reference drug).

Sample	Promastigotes	Cytotoxicity	Intracellular amastigotes	
	IC_50_ (μg/mL) *	CC_50_ (μg/mL) *	IC_50_ *	SI
SFE-CO_2_	16 ± 0.25 ^a^	93 ± 1.52 ^c^	7 ± 0.15	13.28
313 K and, 12.55 MPa				
SFE-CO_2_	27.9 ± 3.9 ^b^	67.5 ± 15 ^d^	13.65 ± 1.25 ^e^	4.94
333 K and, 20.5 MPa				
Compressed propane	29.8 ± 3.8 ^b^	55 ± 4.45 ^d^	22.85 ± 1.85 ^f^	2.41
293 K and, 15.0 MPa				
Compressed propane	23.5 ± 3.2 ^a,b^	43 ± 7.55 ^d^	12.85 ± 0.35 ^e^	3.34
313 K and, 15.0 MPa				
Compressed propane	34.4 ± 7.63 ^b^	91 ± 8.5 ^c^	23.65 ± 1.25 ^f^	3.84
333 K and, 15.0 MPa				
Chloroform extract	15.0 ± 3.0 ^a^	49.5 ± 1.45	13.6 ± 1.0 ^e^	3.64
Pentamidine Isethionate	1.3 ± 0.08	41.48 ± 2.96	0.89 ± 0.12	46.61

* Values represent the mean ± SD of experiments performed in triplicates for antipromastigote activity and evaluation of cytotoxicity, or in duplicates for anti-amastigote activity; SI = CC_50_ for macrophages J774A1/IC_50_ for intracellular amastigotes; Means followed by same lowercase letters did not differ statistically (Tukey test, *p* > 0.05).

The SFE-CO_2 _(313 K; 12.55 MPa) extract could be used to develop a topical phytotherapic for the treatment of the cutaneous leishmaniasis. Topical formulations are excellent alternatives, since drugs used in the antileishmanial therapy are painful and intraperitoneally injected.

Considering the current requirement of the industries about products without organic solvent residues, and people may have allergies to ethanol and other solvents, there is a need to develop extracts from medicinal plants using clean technologies [52]. Therefore, the supercritical fluid extraction employing carbon dioxide may be an appropriate method, due fast production of medicines without toxic residues.

## 3. Experimental

### 3.1. Plant Material

*Piper amalago* L. leaves were collected from the Horto Florestal Dr. Luís Teixeira Mendes in Maringá, Paraná, Brazil. A voucher specimen was deposited in the herbarium of the Department of Botany, University of Maringá (number HUEM 9885). Fresh leaves were dried in an air circulating oven (QUIMIS^®^, model Q-31), at 313 K. After three days, the material was crushed in a knife grinder (Tecnal Marconi^®^, model TE 048, Piracicaba, Brazil) and the resulting powder was classified by size in a system of vibratory sieves (Bertel model 1868, São Paulo, Brazil). Particles with medium diameter of 0.757 mm were used for preparation of the extracts using maceration, supercritical carbon dioxide, and compressed propane.

### 3.2. Extraction Using Supercritical Carbon Dioxide and Compressed Propane

The experiments were performed in a laboratory scale unit. The equipment used was: CO_2_ and propane reservoirs (both technical grade obtained from White Martins – Rio de Janeiro, Brazil), two thermostatic baths, a syringe pump (Teledyne Isco, model 500D – Lincoln, U.S.A) and an extractor with dimensions of 17 × 2 cm, an absolute pressure transducer (Smar, model LD 301 – São Paulo, Brazil) equipped with a portable program (Smar, model HT 201 – São Paulo, Brazil) with an accuracy of ±0.031 MPa, a micrometric valve, and amber glass bottles as collectors. The extractor was loaded with approximately 12 g of powdered sample. The conditions used for extractions with CO_2_ and compressed propane are shown in Table 4. The temperature of the restrictor was kept at 383 K ± 2 K. After a pre-established period, extraction was interrupted for measurement of the extracted mass. The extraction conditions in Table 4 were based on previous extract conditions using both pressurized solvents which led to the alkaloid of interest in the extracts. All conditions of extractions were carried out in duplicate. The conditions of the temperature and pressure for SFE-CO_2_ were based on those described in the literature for alkaloids, which suggested that the extraction of pyrrolidine alkaloid may be possible [30,32,33]. Extraction conditions of compressed propane were based on previous experiments carried out in our laboratory [37,53,54].

**Table 4:** Conditions used to produce *P. amalago* L. leaf extracts with supercritical CO_2_ and compressed propane.

Solvent	Dried plant amount (g)	Temperature (K)	Pressure(MPa)	Density (g/mL)	Flow rate(mL/min)
CO_2_	12.92	313	12.55	0.7349	2
CO_2_	12.57	333	20.50	0.7331	2
Propane	10.09	293	15.0	0.5320	2
Propane	10.52	313	15.0	0.5088	2
Propane	11.07	333	15.0	0.4836	2

### 3.3. General Experimental Procedures

Chromatographic columns with silica gel 60 (70–230 and 230–430 mesh) were used to purify the compounds. The compounds were identified on a (EI) Shimadzu GC/MS 17 A QP 5000, using a DB5 column. ^1^H- and ^13^C-NMR spectra were recorded on a Varian Gemini 2000 BB spectrometer (at 300 and 75 MHz, respectively).

#### 3.3.1. Purification of Alkaloids

Leaves were dried at a temperature of 313 K for 3 days and then powered (250 g). The extract was obtained by maceration at room temperature with ethanol: water (9:1; v/v) (15 × 5 L), filtered and concentrated under vacuum at 313 K. This procedure resulted in a water-soluble phase and a dark residue. The residue was dissolved in chloroform, removed from the round-bottomed flask and transferred to a previously weighed flask. The solvent was evaporated at room temperature, yielding the chloroform extract (21 g). The chloroform extract (12 g) was placed on a vacuum silica gel column (70–230 mesh) and eluted with hexane, hexane-dichloromethane (50:50; v/v), dichloromethane, ethyl acetate and methanol. The ethyl acetate fraction (1 g) was subjected to silica gel column chromatography (40 × 2 cm) using hexane, hexane-chloroform (98:2 to 50:50; v/v), chloroform, chloroform: ethyl acetate (95:5 to 50:50; v/v), ethyl acetate, acetone and methanol, yielding 14 fractions. The fraction F2 (139 mg) was rechromatographed by silica gel column chromatography (20 × 1.5 cm) with hexane-ethyl acetate (50:50; v/v), ethyl acetate and methanol, yielding 50 mg of *N*-[7-(3′,4′-methylenedioxyphenyl)-2(Z),4(Z)-heptadienoyl]pyrrolidine (**1**, Figure 1). Fraction F6 (43 mg) was rechromatographed by silica gel column chromatography (20 × 0.5 cm) (230–400 mesh) with hexane-ethyl acetate (70:30; v/v), ethyl acetate and acetone to yield a subfraction (14 mg). Silica gel column chromatography (10 × 0.5 cm, particle 230–400 mesh) was then applied to this subfraction with hexane-ethyl acetate (50:50; v/v) and ethyl acetate as eluents, yielding 3 mg of *N*-[7-(3′,4′-methylenedioxyphenyl)-2(E),4(E)-heptadienoyl]pyrrolidine (**2**, Figure 1). The compounds were identified by EIMS, ^1^H- and ^13^C-NMR spectral data and by comparison with data available in the literature [7,21].

N-[7-(3′,4′-Methylenedioxyphenyl)-2(Z),4(Z)-heptadienoyl]*pyrrolidine* (**1**). ^1^H-NMR (CDCl_3_) δ: 1.83–1.90 (m, H2′′, H3′′; 4H); 2.40–2.47 (m, H6; 2H); 2.63–2.68 (m, H7; 2H); 3.45 (t, *J* = 6.6 Hz, H1′′; 2H); 3.52 (t, *J* = 6.6 Hz, H4′′; 2H); 5.79 (d, *J* = 11.4 Hz, H2; 1H); 5.90 (s, H1′′′; 2H); 5.91–6.01 (m, H5; 1H); 6.38 (t, *J* = 11.4 Hz, H3; 1H); 6.62 (dd, *J* = 1.6; 7.9 Hz, H6′; 1H); 6.66 (d, *J* = 1.8 Hz, H2′; 1H); 6.72 (d, *J* = 7.8 Hz, H5′; 1H); 7.32–7.42 (m, H4; 1H). ^13^C-NMR (CDCl_3_) δ: 24.52 (C3′′); 26.38 (C2′′); 35.11 (C6); 35.23 (C7); 45.67 (C1′′); 47.07 (C4′′); 100.90 (C1′′′); 108.28 (C2′); 109.02 (C5′); 118.26 (C2); 121.28 (C6′); 128.02 (C4); 135.58 (C1′); 140.64 (C3); 141.81 (C5); 145.78 (C3′); 147.66 (C4′); 165.73 (C1). EIMS *m/z*: 300 (4); 299 (M^+^, 19); 201 (5); 164 (13); 150 (25); 135 (100); 98 (8); 77 (12); 71 (15); 57 (18).

N-[7-(3′,4′-Methylenedioxyphenyl)-2(E),4(E)-heptadienoyl]*pyrrolidine* (**2**). ^1^H-NMR (CDCl_3_) δ: 1.77–1.98 (m, H3′′; 2H); 1.90–1.98 (m, H2′′; 2H); 2.39–2.42 (m, H6; 2H); 2.63–2.68 (m, H7; 2H); 3.40–3.46 (m, H1′′; 2H); 3.48–3.56 (m, H4′′; 2H); 5.90 (s, H1′′′; 2H); 6.06 (d, *J* = 15.0 Hz, H2; 1H); 6.06 (dd, *J* = 6.6, 15.0 Hz, H5, 1H); 6.19 (dd, *J* = 10.5, 15.0 Hz, H4; 1H); 6.61 (dd, *J* = 1.6; 7.8 Hz, H6′; 1H); 6.66 (d, *J* = 1.8 Hz, H2′; 1H); 6.72 (d, *J* = 7.8 Hz, H5′; 1H); 7.25 (dd, *J* = 10.5, 15.0 Hz, H3; 1H). ^13^C-NMR (CDCl_3_) δ: 24.57 (C3′′); 26.34 (C2′′); 35.18 (C7); 35.27 (C6); 46.09 (C4′′); 46.68 (C1′′); 100.99 (C1′′′); 108.36 (C5′); 109.04 (C2′); 120.58 (C2); 121.38 (C6′); 129.54 (C4); 135.35 (C1′); 141.7 (C5); 142.1 (C3); 145.91 (C3′); 147.76 (C4′); 165.73 (C1). EIMS *m/z*: 300 (4); 299 (M^+^, 19); 201 (5); 164 (13); 150 (25); 135 (100); 98 (8); 77 (12); 71 (15); 57 (18).

### 3.4. HPLC Analysis

#### 3.4.1. Reagents and Chemicals

HPLC grade acetonitrile was supplied by J. T. Baker SOLUSORB^®^ (Xalostoc, Mexico). Ultrapure water was obtained from a GEHAKA purification system (São Paulo, SP). Acetic acid was analytical grade (CAQ, Diadema, SP). *N*-[7-(3′,4′-methylenedioxyphenyl)-2(Z),4(Z)-heptadienoyl]pyrrolidine (**1**) used an external standard, and *N*-[7-(3′,4′-methylenedioxyphenyl)-2(E),4(E)-heptadienoyl]pyrrolidine (**2**), used to identify the corresponding peak in the extracts, were previously isolated from *P. amalago* L.

#### 3.4.2. Sample Preparation

The extract was prepared according to the adapted method from Felipe *et al.* [44]. The leaves of *P. amalago* L. were extracted only with chloroform through the maceration process for five days, and then filtered and dried at room temperature. Chloroform was chosen for the method, because the pyrrolidine alkaloids of this plant were more soluble in this solvent, as related in the literature [55]. This procedure was carried out in duplicate.

#### 3.4.3. Chromatographic Conditions

The major alkaloids were quantified using a Waters 1525 Binary HPLC Pump (Waters, Milford, MA, USA) equipped with a manual injection valve with a loop of 20 μL, and a Waters 2489 UV/visible detector, controlled by Waters Breeze 2 Software. Chromatographic separations were carried out in a YMC Pack Pro C18 column (150 × 4.6 i.d.) packed with 5 μm particles of 12 nm porosity size at 298 K. The mobile phase used was 58% of acetonitrile and 42% of water containing 1% acetic acid, at flow rate of 1 mL/min for 10 minutes, changing to 100% of acetonitrile at 11 min to 15 min. The detection of the compounds was at 260 nm. The solutions of the SFE and chloroform extracts were prepared in acetonitrile at 300 μg/mL. The solutions were filtered through a non-sterile 0.45 μm membrane filter (Millipore, São Paulo, Brazil). A 20 μL volume of each sample was manually injected into the HPLC, and the analysis was carried out in triplicate. The data were evaluated by analyses of variance and the Tukey test using GraphPad Prism 5.0 (San Diego, CA, USA). Differences were considered statistically significant when the *p* value < 0.05.

#### 3.4.4. Validation Parameters

##### 3.4.4.1. Linearity

The linearity of the alkaloid calibration curve was determined by the external standard method. A stock standard solution of 1,844 μg/mL in acetonitrile was prepared, and diluted to the concentrations of 184.4, 138.3, 92.2, 46.1, and 23.05 μg/mL. The solutions were filtered through a 0.45 μm membrane filter (Millipore). Three analyses were carried out for each solution. The calibration curves were obtained by plotting the area ratios of the alkaloid *versus* analyte concentration.

##### 3.4.4.2. Precision

In order to evaluate the precision, three concentration levels (184.4, 92.2, and 23.05 μg/mL) were analyzed in triplicate. The precision was expressed as relative standard deviation (% RSD) of the alkaloid concentrations. The repeatability was determined on the same day and the intermediate precision was examined for two non-consecutive days.

##### 3.4.4.3. Accuracy

The accuracy was determined based on the recovery test, analyzing the mixture prepared by adding the standard solutions at the concentration levels 184.4, 92.2 and 23.05 μg/mL to the extract prepared by maceration with chloroform, containing a known amount of the analyte. Three determinations were carried out for each solution. The percentage recovery was calculated as [(C1 − C2/C3) × 100)], where C1 is the concentration of the analyte added to the extract, C2 is the concentration of the extract and C3 is the concentration of the standard.

##### 3.4.4.4. Limit of Detection and Quantification

The limit of detection and the limit of quantification were calculated according to the expressions 3σ/*S*, and 10 σ/*S*, respectively, where σ is the standard deviation of the interceptor and S is the slope of the calibration curve.

### 3.5. Evaluation of the Extracts Against the Promastigote and Intracellular Amastigote Forms of L. amazonensis

#### 3.5.1. Parasites

In this study, it was used a strain of *L. amazonensis* (MHOM/BR/77/LTB0016). Parasites were isolated from infected mice and maintained as promastigotes through weekly passages in Schneider medium (Sigma^®^ Saint Louis, MO, USA) supplemented with 10% of fetal bovine serum, penicillin (100 UI/mL), and streptomycin (100 μg/mL) at 26 °C.

#### 3.5.2. Cells

J774A1 macrophages were cultured in cell culture flasks of 25 cm^2^ in RPMI medium pH 7.2 supplemented with 10% fetal bovine serum, and incubated at 37 °C under an atmosphere of 5% CO_2_. Macrophage cultures were maintained by passages every two or three days, according to ATCC.

#### 3.5.3. Animals

Male BALB/c mice (25–30 g) were kept in a 12 h light/dark cycle in a temperature-controlled room with free access to water and food. The experimental protocol was submitted to and approval by Fiocruz Ethical Committee on Animal Use (CEUA-Fiocruz protocol number LW-7/10).

### 3.5.4. Stock Solutions

The extracts were dissolved in dimethyl sulfoxide (DMSO, Sigma^®^). Stock solutions of 10 mg/mL of the extracts were prepared in DMSO. Assay concentrations were prepared in culture medium used in the experiments, as indicated in each case.

#### 3.5.5. Antileishmanial Activity Against Promastigotes

The method used to determine antipromastigote activity was adapted from Denizot and Lang [56]. Promastigotes were adjusted to a concentration of 1 × 10^6^ cells/mL in Schneider medium (supplemented with 10% de fetal bovine serum, penicillin (100 UI/mL), and streptomycin (100 μg/mL), and incubated at 26 °C for 72 h with extracts (3.125–200 μg/mL) (0.5% DMSO was used to dissolve the highest concentration of the samples). Promastigotes in culture medium supplemented as above, but without test compounds, were used as the negative control. Pentamidine isotionate was used as the positive control. The antileishmanial activity was evaluated by adding in each well 22 μL of MTT [3-(4,5-dimethylthiazol-2-yl)-2,5-diphenyltetrazolium bromide] at 5 mg/mL (Sigma^®^). After 2 h, 80 μL of DMSO was added. The optical density was determined at a wavelength of 570 nm in microplate reader (µQuant Bio-Tek Instruments^®^, Winooski, VT, USA). The inhibition percentage was estimated by the comparison with non-treated control cultures. The assays were carried out in triplicate in 96-well plates (Costar^®^, New York, NY, USA).

#### 3.5.6. Cytotoxicity Evaluation

The cytotoxicity test was adapted from Mendez [57]. The toxicity of the compound was analyzed using continuous J774A1 macrophage lineage. The macrophages at 2 × 10^6^ cells/well in RPMI culture medium (pH 7.2, supplemented with 10% fetal bovine serum) were incubated with extracts (50–400 μg/mL) for 72 h at 37 °C under 5% CO_2_ in 96-well plates. Cells in culture medium plus DMSO (0.5%) were used as control of viability. Pentamidine isethionate was used as the reference. After removing the supernatant, viable cells were quantified by adding MTT (200 μL, 5 mg/mL) in phosphate buffer saline (PBS). After 2 h, the supernatant was removed and DMSO (100 μL) was added in each well. The optical density was determined at wavelength of 570 nm in the microplate reader. The percentage of viable cells was calculated relative to the control cells. The tests were carried out in triplicate.

#### 3.5.7. Antileishmanial Activity Against Intracellular Amastigotes

BALB/c mice macrophages were obtained by peritoneal lavage with 5 mL of cold RPMI medium (Sigma^®^). The cell suspension (2 × 10^6^ macrophages/mL) was applied in Labtek chambers (Nunc^®^, New York, NY, USA) and incubated for 1 h at 37 °C, 5% CO_2_. Then, the cultures were washed with (PBS) at 37 °C for removal of non-adherent cells. The remaining cells were incubated at 37 °C, 5% CO_2_ with stationary phase promastigotes of *L. amazonensis* at a ratio of 3:1. After 3 h, the chambers were washed again to remove free parasites and incubated with extracts (12.5–200 μg/mL) at 37 °C, 5% CO_2_ for 72 h. Infected cells with DMSO (0.5%) were used as negative control. Pentamidine isethionate was used as the positive control. The anti-amastigote activity was analyzed microscopically by counting at least 100 macrophages per sample, after staining cells with haematological system Instant Prov (New Prov^®^, Curitiba, Brazil) [58]. The experiments were performed twice in duplicate. Results were expressed as ratio of infection (IF) using the following formula:


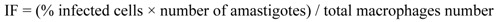


IF = (% infected cells × number of amastigotes) / total macrophages number

#### 3.5.8. Assay for Nitric Oxide Production

After 72 h of incubation with the most active extract against the amastigote forms of *Leishmania*, the supernatants of infected macrophages were collected for quantification of secreted nitric oxide by determining the nitrite concentration using the Griess assay. Griess reagents (1% sulfanilamide/0.1% naphthylethylenediamine dihydrochloride/3% of phosphoric acid) were mixed 1:1 with supernatant, and left to stand for 5 min at room temperature. The absorbance was determined at 470 nm in a microplate reader. The nitrite concentration was calculated from a standard curve of sodium nitrite (10 to 50 μM). The experiments were performed twice in duplicate [59,60].

#### 3.5.9. Statistical Analysis

The antileishmanial activity was expressed as growth inhibition. Logarithm regression analysis was performed in order to obtain the values of IC_50_ (concentrations that inhibit growth by 50% of promastigotes and amastigotes) and CC_50_ (cytotoxic concentration for 50% macrophages). These data were evaluated by variance analyses and Student´s t test by using GraphPad Prism 5.0 (San Diego, CA, USA). It was considered significant difference when the *p* value <0.05.

## 4. Conclusions

This paper has described the development and validation of a HPLC method for quantification of the pyrrolidine alkaloid *N*-[7-(3′,4′-Methylenedioxyphenyl)-2(Z),4(Z)-heptadienoyl]pyrrolidine (**1**) in extracts of *P. amalago* L. leaves. The HPLC method demonstrated linearity, precision and accuracy in the concentration range for the alkaloid, complying with regulatory requirements. Therefore, the method contributed to the quantitative control of the major compound in the extracts. Supercritical CO_2_, compressed propane, and maceration methods were examined with respect to pyrrolidine alkaloid content, using the validated HPLC method. The results indicated that extraction efficiencies achieved using the maceration method are higher than those attained by applying SFE-CO_2_ and compressed propane, however, the results for supercritical carbon dioxide at 313 K and 12.55 MPa showed that this is a suitable solvent for the extraction of the alkaloid due to the lower temperature and pressures and the shorter time required compared to the use of propane and the organic solvent. Furthermore, SFE-CO_2_ (313 K; 12.55 MPa) produced an extract with less undesired components, indicating that this method enhanced extraction selectivity compared to the conventional technique. The SFE-CO_2_ (313 K; 12.55 MPa) extract showed the highest antileishmanial activity, with strong activity on promastigotes, selective action against intracellular amastigotes, and the highest SI. This present study may be important for development of new phytomedicines for the treatment of the cutaneous leishmaniasis.
